# Early and mid-term outcomes of simultaneous thoracic endovascular stent grafting and combined resection of thoracic malignancies and the aortic wall

**DOI:** 10.1007/s11748-018-1003-1

**Published:** 2018-09-01

**Authors:** Seijiro Sato, Atsuhiro Nakamura, Yuki Shimizu, Tatsuya Goto, Akihiko Kitahara, Terumoto Koike, Takeshi Okamoto, Masanori Tsuchida

**Affiliations:** Division of Thoracic and Cardiovascular Surgery, Niigata University Graduate School of Medical and Dental Sciences, 1-757 Asahimachi-dori, Chuo-ku, Niigata, Niigata 951-8510 Japan

**Keywords:** Lung cancer, Thoracic malignancy, Thoracic endovascular stent grafting, One-stage procedure, Combined aortic resection

## Abstract

**Objectives:**

To aim of this study was to clarify the safety of simultaneous thoracic aortic endografting and combined resection of the aortic wall and thoracic malignancy in a one-stage procedure over the early and mid-term periods.

**Methods:**

From March 2013 to December 2017, 6 patients underwent aortic endografting followed by one-stage en bloc resection of the tumor and aortic wall. Thoracic surgeons and cardiovascular surgeons discussed predicted tumor invasion range and resection site, stent placement position, stent length and size, and the surgical procedure, taking into account the safe margin.

**Results:**

The proximal site of aortic endografting was the: aortic arch in 2 cases (subclavian artery (SCA) occlusion in one, and SCA fenestration in one); distal arch just beneath the SCA in 2; descending aorta in 2. Pulmonary resection involved lobectomy in 2 patients, pneumonectomy in 2, and completion pneumonectomy in 1. Aortic resection was limited to the adventitia in 2 cases, extended to the media in 3, and extended to the intima in 1. An endograft-related complication, external iliac artery intimal damage requiring vessel repair, was observed in one case. No complications associated with aortic resection were observed. Two postoperative complications of atrial fibrillation and chylothorax developed. There were no surgery-related deaths. During follow-up, no late endograft-related complications such as migration or endoleaks occurred.

**Conclusions:**

Early and mid-term outcomes of stent graft-related complications are acceptable. Simultaneous thoracic aortic endografting and combined resection of the aortic wall and thoracic malignancies are feasible in one stage on the same day.

## Introduction

Combined resection of tumor and aortic wall for thoracic malignancy is highly invasive and has been a challenging procedure for thoracic surgeons. The Japanese Association for Thoracic Surgery conducted a survey of lung cancer surgery, which was published as an annual report [1]. In 16 (0.042%) of 38,085 lung cancer patients in 2014, combined resection of the lung and an infiltrated aortic wall was performed for locally advanced T4 lung cancer. Previously, this procedure usually implied the need for cardiopulmonary bypass (CPB) or extra-anatomical bypass, which are associated with high morbidity and mortality [2–6]. With the advent of minimally invasive endovascular therapy with thoracic endovascular aortic repair (TEVAR) in recent years, resection of the aorta may be performed with a less invasive approach than using CPB. The safety of simultaneous thoracic aortic endografting and combined resection of the aortic wall and thoracic tumor on the same day over the early and mid-term period was evaluated.

## Methods

This was a retrospective, single-center study. The surgical approach was approved by the Institutional Review Board of Niigata University Hospital (No. 2658), and informed consent was obtained from all the patients. From March 2013 to December 2017, 6 patients underwent endovascular stent grafting followed by en bloc resection of the tumor and aortic wall, and their data were retrospectively reviewed. The patients’ characteristics are shown in Table 1.

**Table 1:** Patients’ characteristics

Patient no.	Sex	Age (years)	Tumor size (mm)	Preoperative therapy	Histology	cTNM	Site of aortic invasion	Endograft		
								Type	Length (mm)	Diameter (%)
1	Male	67	95	None	Pleomorphic carcinoma	T4N1M0	Descending aorta	Valiant–Valiant	200–150	126–126
2	Male	76	68	None	Squamous cell carcinoma	T4N1M0	Descending aorta	Valiant–Valiant	100–100	115–125
3	Male	60	70	None	Adenocarcinoma	T4N0M0	Aortic arch	Valiant–Valiant	150–150	123–125
4	Male	56	45	Chemoradiation	NOS	T4N0M0	Distal arch	Relay–Relay	150–100	115–125
5	Male	77	47	Radiation	Adenocarcinoma	T4N0M0	Distal arch	Relay–TX2	150–80	121–120
6	Male	61	25	None	Leiomyosarcoma		Descending aorta	Valiant–Valiant	150–150	117–126

*NOS* not otherwise specified, *Valiant* Valiant (Medtronic Vascular, Santa Rosa, CA, USA), *Relay* Relay (Bolton Medical, Sunrise, FL, USA), *TX2* TX2 (COOK Medical, Bloomington, IN, USA)

### Preoperative staging

In cases of non-small cell lung cancer (NSCLC), assessments of the preoperative computed tomography (CT) of the chest and abdomen, CT or magnetic resonance imaging (MRI) of the brain, bone scan, or integrated positron emission tomography with CT (PET-CT) were performed. MRI of the spine was performed when spinal infiltration was suspected. If mediastinal node involvement was suspected, the mediastinum was assessed by endobronchial ultrasound-guided fine-needle aspiration. Radiological findings suggestive of aortic infiltration were obliteration of the fat plane between the aorta and the tumor, aortic compression, or contact by the tumor of more than a third of the aortic circumference. When the approach to the left subclavian artery was considered necessary, magnetic resonance angiography (MRA) was performed before surgery to confirm that the following were not present: (1) dominant left vertebral artery (VA); (2) absent or diminutive or occluded right VA; (3) no communication of bilateral VAs. If not, axillo–axillo bypass surgery was considered before the thoracic endovascular stent grafting. Induction chemoradiotherapy was only performed in patient 4 because of a mass in the left upper lobe with probably wide invasion of the adjacent distal aorta and spine, which was described in detail previously [7]. Tumors were classified according to the seventh edition of the TNM classification of malignant tumors [8].

In cases of sarcoma, postoperative radiotherapy was performed with CT of the chest and abdomen.

### Surgical technique

Before endovascular stent grafting, CT angiography was performed from the supra-aortic vessels to the common femoral arteries to assess vessel size and anatomy and to delineate the maximum possible area of aortic invasion. In all cases, one-stage procedures were performed under general anesthesia, using a retrograde femoral approach. Two endovascular stent grafts of the same brand were selected, about 10% larger than the native aorta in each case.

First, in the lateral position, the infiltrated aortic wall was confirmed by careful palpation, and then, the proximal and distal ends of the tumor infiltrating aortic wall were marked with surgical clips to guide endovascular stent grafting by exploratory thoracotomy or thoracoscopy. Second, with the patient in the supine position, heparinization was administered to a target-activated coagulation time (ACT) of about 200 s. Proximal and distal landing zones that consisted of 4 cm of healthy aorta were targeted for sufficient endovascular stent graft stability. Third, after neutralization with protamine, tumor resection was performed en bloc and included resection of the involved aortic wall, lung, chest wall, and vertebral body when necessary in the lateral position. For NSCLC, anatomic resection plus systematic lymphadenectomy was performed.

### Postoperative follow-up

After discharge from the hospital, patients were followed-up in our hospital outpatient department at 3-month intervals. Patients were routinely evaluated for tumor recurrence or metastasis and complications after endovascular stent grafting, such as migration and endoleaks, every 3–6 months by chest contrast-enhanced CT during the first 2 years after surgery and every 6 months thereafter.

## Results

**Fig. 1:**
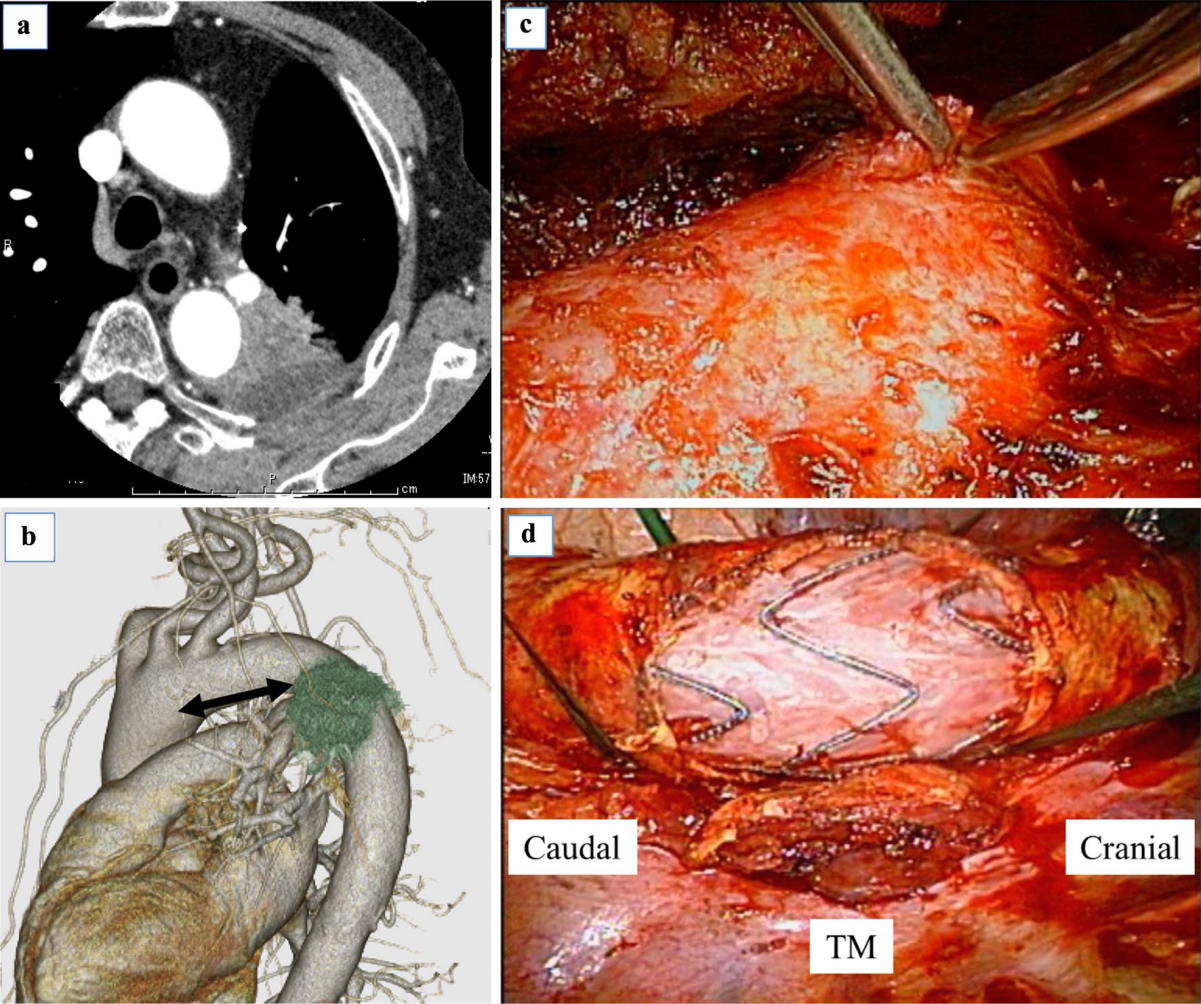
**a** CT shows a mass of the left lower lobe invading the aortic arch, obliteration of the fat plane between the aorta and the tumor, and contact by the tumor of more than a third of the aortic circumference. **b** Three-dimensional CT shows the positional relationship between the aortic arch and the tumor. The arrows indicate the distance of the proximal 4-cm-long margin of the healthy aorta from the tumor that involves the left subclavian artery. **c** Intraoperative view shows resection of the aortic wall, sparing the media in patient 5. **d** Intraoperative view shows that the stent can be seen through the defect of the aortic wall in patient 6. *TM* tumor

The median follow-up period after surgery was 17.6 months (range 8–25 months). The median tumor size was 57.5 mm (range 25–95 mm). Histological diagnoses were primary lung cancer in 5 patients: 2 with adenocarcinoma, 1 with squamous cell carcinoma, 1 with pleomorphic carcinoma, and 1 with cancer not otherwise specified. The other patient had leiomyosarcoma derived from the descending aorta. Patient 5 had multiple metachronous primary lung cancers and underwent left upper lobectomy for lung adenocarcinoma 14 years earlier. He presented with a new lesion at segment 6 of the left lower lobe 3 years earlier. Stereotactic body radiotherapy (SBRT) with 54 Gy in 4 fractions was administered to the tumor. However, because the tumor gradually increased, he underwent salvage surgery for recurrence after SBRT. Intraoperatively, the tumor involved the distal aortic arch, and the operation then ended with an exploratory thoracotomy. He was referred to our hospital for surgical resection of a T4 recurrent lung cancer infiltrating the aorta after SBRT (Fig. 1a, b).

The operative and postoperative results are summarized in Tables 2 and 3, respectively. Pulmonary resection involved lobectomy in 2 patients, pneumonectomy in 2 patients, and completion pneumonectomy in 1 patient. Aortic resection was limited to the adventitia in 2 cases, extended to the media in 3, and extended to the intima in 1 (Fig. 1c, d). With the exception of patient 4, reinforcement of the aortic wall defect was not performed. The duration of endovascular stent grafting was 88.5 min (range 49–149 min). An additional procedure of thoracic aortic endografting was required in 2 patients. For the purpose of securing a sufficient margin from the tumor and landing zone, left subclavian artery (LSCA) occlusion was needed in patient 3, and a fenestrated stent graft that would open the LSCA was used in patient 5. Regarding intraoperative complications related to endovascular stent grafting, patient 1 had subintimal injury of the external iliac artery, but the others had no complications. R0 resection was achieved in all patients except patient 3. In patient 3, because the tumor had a wide invasion range from the aortic arch to the subclavian artery and the distal arch, and invasion into the vertebral body was strongly suspected; incomplete aortic wall resection was performed. Postoperative complications developed in 3 patients (2 had supraventricular arrhythmias treated by medication, and 1 had chylothorax treated by no oral intake and pleurodesis), but no severe complications occurred. Adjuvant radiation therapy was given to 3 patients. Taking into account age and performance status, adjuvant radiation therapy was administered in cases where invasion into the adjacent tissue was suspected or in incomplete resection cases. In patient 2, a large tumor with pleural invasion showed tight adhesions to the chest wall widely. In patient 3, incomplete surgical resection was performed. In patient 6, the tumor involved the adjacent vertebral body. The radiation field was mainly the chest wall in patient 2 and the thoracic vertebral body in patient 6.

**Table 2:** Operative procedures and perioperative results

Patient no.	Aortic endografting		En bloc resection						Duration		Blood loss (ml)
	Proximal landing zone	Additional procedure	Lung	Chest wall	Vertebrectomy	Aorta			Total operation	TEVAR	
						Resection	Extent	Circumference			
1	Distal arch		Pneumonectomy			Adventitia	40 mm	1/3	7 h 30 min	2 h 29 min	190
2	Descending aorta		Pneumonectomy			Adventitia	40 mm	1/3	7 h 41 min	49 min	860
3	Aortic arch	SCA occlusion	Upper lobectomy			Adventitia–media	NA	NA	6 h	1 h 36 min	760
4	Distal arch		Upper lobectomy	Rib 3–5	Total T3–5	Adventitia–media	40 mm	1/4	18 h 10 min	1 h 21 min	1430
5	Aortic arch	SCA fenestrated	Completion pneumonectomy			Adventitia–media	50 mm	1/3	7 h 21 min	1 h 55 min	595
6	Descending aorta		None		Hemi T7–9	Adventitia–media–intima	45 mm	1/2	10 h 16 min	77 min	1260

*h* hours, *min* minutes, *SCA* subclavian artery, *NA* not available, *TEVAR* thoracic endovascular aortic repair

**Table 3:** Postoperative results and follow-up

Patient no.	(y)pTNM	Postoperative therapy	Complication	DFS (months)	Recurrence	Status
1	T3N1M0	None	Subintimal injury of EIA, atrial flutter	11	Distant (bone)	Dead
2	T4N1M0	Radiation	Atrial fibrillation	6	Locoregional (pleural dissemination)	Dead
3	T4N0M0	Radiation	None	11	Local (aorta)	Dead
4	T0N0M0	None	Chylothorax	15	No	Alive
5	T4N1M0	None	None	12	No	Alive
6		Radiation	None	3	Local (vertebral body)	Dead

*NOS* not otherwise specified, *DFS* disease-free survival, *EIA* external iliac artery

During the follow-up period, 4 patients died from cancer-related causes: 2 patients had local recurrence; 1 patient had locoregional recurrence; 1 patient had distant recurrence. In the cases of R0 resection, no evidence of recurrence was found at the region of aortic wall resection. No late endograft-related complications, such as stenosis, migration, endoleak, or deformation, were observed.

## Discussion

Surgical resection of a T4 lung cancer invading the aorta or sarcoma originating from the aorta has been challenging for thoracic surgeons. This study described the off-label use of endovascular thoracic stent grafts commonly used for aortic aneurysms, dissection, and rupture in 6 oncologic cases and presented the early and mid-term outcomes of one-stage surgery. Previously, if combined aortic and pulmonary resection was required in patients with locally advanced lung cancer, cardiopulmonary bypass (CPB) or temporary extra-anatomical aortic bypass was needed to prevent organ ischemia [2–6]. However, the use of CPB during resection of locally invasive lung cancer has been associated with concerns about several problems. CPB is known to impair coagulation, and systemic heparinization required for CPB can increase the bleeding risk. In addition, CPB stimulates a proinflammatory response and has been shown to induce temporary immunosuppression [9, 10]. Furthermore, although there have been no definitive studies, if tumor cells are introduced into the extracorporeal circuit, there is a theoretical risk of hematogenous dissemination [11]. In 2008, Marulli and colleagues first reported the procedure of resection of lung metastasis invading the aortic wall using an off-label endovascular stent graft without CPB [12]. Since then, aortic wall resection under thoracic endovascular stent grafting has been reported as a less invasive procedure [7, 13–20]. So far, no endovascular stent graft-related perioperative and late complications have been reported, unlike with CPB.

Accurate evaluation of tumor infiltration depth into the aortic wall has been difficult. ECG-gated CT and cine dynamic MRI are useful diagnostic tools for aortic infiltration of thoracic tumors [21, 22]. However, evaluation of major structures with minimal-to-no motion such as the distal aortic arch is limited with these devices. Therefore, a definite diagnosis should be made by exploratory thoracoscopy or thoracotomy. A one-stage procedure can confirm aortic wall infiltration of thoracic malignancy directly, and it is possible to place an endovascular stent graft in cases where it is necessary. In most reported cases, lung cancer surgery in combination with resection of the infiltrated aortic wall was performed several days after thoracic endovascular stent grafting [12–15, 17–20]. However, the one-stage procedure in the present study was performed without stent graft-related complications, such as paraplegia, stroke, endoleak, or stent migration, in the perioperative period. Marulli and colleagues also suggested that additional anesthesia and overtreatment in cases that do not have deep aortic infiltration were avoided in a one-stage procedure [16]. So far, the optimal timing between endovascular stent graft deployment and aortic wall resection is unclear.

As regards the proximal and distal margins of the healthy aorta, a length of at least 4 cm has been reported to ensure safety and stent graft stability [14, 16]. According to the previous reports, the present series targeted the same length, which was why two endovascular stent grafts were needed in each case. Walgram and colleagues reported that a length of at least 2 cm was needed for the proximal and distal landing zones, because the length of the aortic segment covered by the stent should be kept as short as possible to occlude as few intercostal arteries as possible [20]. It has been unclear to what extent that the aortic wall can be resected. With respect to the decision as to whether to perform full-thickness or partial-thickness resection of the aortic wall, the aortic wall was dissected with a non-invasive and peelable layer based on the intraoperative findings. Collaud and colleagues reported that, when a partial thickness of the aortic wall and less than one-half of the circumference were resected, no stent graft-related complications were encountered [14]. However, they had a case that developed bulging of the endograft out of the aortic wall defect after full-thickness wall resection of over 5 cm. Marulli and colleagues advocated that resection of a partial thickness of the aortic wall or less than one-half of the circumference was possible without covering the aortic defect [16]. Walgram and colleagues suggested that full-thickness aortic wall resection might be possible in up to a third of the circumference [20]. As preparation for this surgical procedure, it is very important to keep in mind the management of intraoperative bleeding from the resected aortic wall. We think that massive bleeding does not occur with long (at least 4 cm) proximal and distal margins of healthy aorta, but the possibility of minor endoleaks has to be kept in mind. Therefore, femoral arteriovenous access is secured, so that an occlusion balloon for hemostasis or CPB can be used immediately. No previous studies reported this issue, but further study of the management strategy will be necessary. The necessity of aortic wall defect buttressing remains unknown. Marulli and colleagues carefully considered whether to suture a synthetic patch or an omental flap onto the aortic wall defect in cases of full-thickness aortic resection or resections extending over one-half of the circumference [16]. Walgram and colleagues suggested that, when the full thickness of the aortic wall was resected, reinforcement of the aortic wall defect with a xenopericardial patch might be required to avoid bulging of the endograft out of the aortic wall defect [20]. In this series, to ensure a sufficient margin away from the tumor and landing zone, 2 endovascular stent grafts were used, and the aortic wall defect was doubly lined in all the cases. Therefore, except for patient 4 who underwent left upper lobectomy combined with resection of the infiltrated aortic wall and vertebral body, the aortic wall defect was not buttressed, even though the full-thickness of the aortic wall over 4 cm and one-half of the circumference were resected in one case. As mentioned above, several studies reported that aortic wall defect buttressing is not necessary except under certain conditions. However, if these patients survive for a long time, the possibility of severe complications, such as stent migration and endoleaks, remains. After endovascular stent grafting for thoracic aortic aneurysm or abdominal aortic aneurysm in our institution, additional stent grafts are placed when these serious postoperative complications occur. Therefore, similar treatment will be performed even after this novel procedure. Therefore, long-term imaging follow-up, such as with contrast-enhanced CT, may be necessary.

Long-term outcomes after this procedure involving the off-label use of endovascular stent grafts are important. In particular, stent graft-related events, such as graft migration and type 1 endoleak, have a high risk of a fatal outcome. Recently, some researchers reported the long-term outcomes of endovascular repair of abdominal aortic aneurysms. In the OVER study reported by Lederle and colleagues, of 444 patients with endovascular repair, 6 developed rupture of an abdominal aortic aneurysm, of which 4 occurred more than 5 years after the repair [23]. Of the six patients with rupture, graft migration and a large type 1 endoleak were observed in one patient each. The Japanese Committee for Stentgraft Management report, which included 3,124 patients, reported stent graft migration and type 1 endoleaks at 6 months after repair in 3 patients (0.1%) and 13 patients (0.5%), respectively [24]. Based on the above, longer follow-up is necessary to evaluate the full potential of this procedure.

Regarding the cost of off-label stent graft treatment, two off-label stent grafts were used for each patient, costing approximately 3 million yen per patient. A detailed statement of medical expenses for this novel surgical approach was created for each patient, and then, all but one case were covered by National Health Insurance (NHI) so far. In addition, we have been approved for medical expenses subsidy application for this surgical procedure and have been paid 2 million yen per surgery from our institution. The remaining costs have been paid by our hospital unless NHI covered this treatment. Thus, consultation with payment funds in each area and institution seems to be necessary.

## Conclusions

Thoracic endovascular stent grafting may facilitate the resection of malignant tumors infiltrating the aortic wall. The early and mid-term outcomes, especially in terms of stent graft-related complications, are acceptable. Simultaneous thoracic endovascular stent grafting and combined resection of the aortic wall and thoracic malignancy are feasible in one stage on the same day. Prior to surgery, thoracic surgeons should share information with cardiovascular surgeons to ensure the safety of this procedure.
